# A Pathological and Physiological Essay on Hereditary Diseases. With an Appendix on Intermarriage, and on the Inheritance of the Tendency to Moral Depravities and Crimes

**Published:** 1844-01

**Authors:** 


Art. VIII.—*A Pathological and Philosophical Essay on Hereditary Diseases. With an Appendix on Intermarriage, and on the Inheritance of the Tendency to Moral Depravities and Crimes.* By J. H. Steinau, m.d., of the Royal Medical College, Berlin.—*London*, 1843. 8vo, pp. 52.

This *brochure* may be regarded as a second edition of an essay which was published some years since in Germany; where, the author informs us, it met with a very favorable reception. It contains a good summary of the recorded opinions and experience of a large number of celebrated physicians, and some interesting observations made by Dr. Steinau himself; but we do not find anything very original in his mode of viewing the subject. As a lucid compendium of the present state of our knowledge, however, it may be read with advantage. We do not venture to anticipate that *any* improvements in physiological knowledge will ever teach us how the influence of the *male* parent is exerted upon the germ, so as to impress on it the tendency to repeat, in its own body when fully developed, and in the germ of a succeeding generation, certain peculiar disordered actions; any more than we expect to be able to find out why a particular feature or other peculiarity of his own is repeated in his offspring: or, to take a more general case, state why the first embryonic cell, which *seems* to be alike in all animals, should develop itself into the form of the genus Homo, rather than into any other. These are mysteries which we cannot solve in our present state of existence; and we can only account for them by regarding them as instances of a general law. The influence of the female parent, upon whom depend the nutrition and support of the germ from its earliest introduction into the ovum, up to the time when it can maintain itself, is more easily accounted for; and knowing what a remarkable effect the state of the mind has upon the processes of nutrition and secretion in the parent, we can scarcely wonder at any result which manifests itself in the offspring. We think that the subject of hereditary disease would be best considered as a branch of the more general inquiry into hereditary propagation of corporeal forms and mental characters in general; and we would suggest to Dr. Steinau the prosecution of his inquiries (a continuance of which he leads us to expect, if time and opportunity be afforded to him,) on this more extended basis, the importance of which, from various intimations in his present essay, we are sure that he fully appreciates.

